# Supplementary material for: Resolving the full spectrum of human genome variation using Linked-Reads
Source: Genome Res. 2019 Apr;29(4):635–45. doi: 10.1101/gr.234443.118 (PMC6442396; doi:10.1101/gr.234443.118)
Supplement: Supplemental Material [file supp_gr.234443.118_Supplemental_longranger-2.2.zip › longranger-2.2.tar.bz2!/lr2.2-public/lib/stan/cmdstan/stan/doc/api/html/rev_2functions_2pow_8hpp_source.html]

Stan: src/stan/agrad/rev/functions/pow.hpp Source File


|  |  |
| --- | --- |
|  | Stan  2.5.0  probability, sampling & optimization |


- Main Page
- Related Pages
- Namespaces
- Classes
- Files

- File List
- File Members

All Classes Namespaces Files Functions Variables Typedefs Enumerations Enumerator Friends Macros Pages

- src
- stan
- agrad
- rev
- functions

pow.hpp

Go to the documentation of this file.

1 #ifndef STAN\_\_AGRAD\_\_REV\_\_FUNCTIONS\_\_POW\_HPP

2 #define STAN\_\_AGRAD\_\_REV\_\_FUNCTIONS\_\_POW\_HPP

3

4 #include <cmath>

5 #include <stan/agrad/rev/var.hpp>

6 #include <stan/agrad/rev/internal/vv\_vari.hpp>

7 #include <stan/agrad/rev/internal/vd\_vari.hpp>

8 #include <stan/agrad/rev/internal/dv\_vari.hpp>

9 #include <stan/agrad/rev/functions/sqrt.hpp>

10 #include <stan/agrad/rev/operators/operator\_multiplication.hpp>

11 #include <boost/math/special\_functions/fpclassify.hpp>

12

13 namespace stan {

14  namespace agrad {

15

16  namespace {

17  class pow\_vv\_vari : public op\_vv\_vari {

18  public:

19  pow\_vv\_vari(vari\* avi, vari\* bvi) :

20  op\_vv\_vari(std::pow(avi->val\_,bvi->val\_),avi,bvi) {

21  }

22  void chain() {

23  if (unlikely(boost::math::isnan(avi\_->val\_)

24  || boost::math::isnan(bvi\_->val\_))) {

25  avi\_->adj\_ = std::numeric\_limits<double>::quiet\_NaN();

26  bvi\_->adj\_ = std::numeric\_limits<double>::quiet\_NaN();

27  } else {

28  if (avi\_->val\_ == 0.0) return; // partials zero, avoids 0 & log(0)

29  avi\_->adj\_ += adj\_ \* bvi\_->val\_ \* val\_ / avi\_->val\_;

30  bvi\_->adj\_ += adj\_ \* std::log(avi\_->val\_) \* val\_;

31  }

32  }

33  };

34

35  class pow\_vd\_vari : public op\_vd\_vari {

36  public:

37  pow\_vd\_vari(vari\* avi, double b) :

38  op\_vd\_vari(std::pow(avi->val\_,b),avi,b) {

39  }

40  void chain() {

41  if (unlikely(boost::math::isnan(avi\_->val\_)

42  || boost::math::isnan(bd\_)))

43  avi\_->adj\_ = std::numeric\_limits<double>::quiet\_NaN();

44  else {

45  if (avi\_->val\_ == 0.0) return; // partials zero, avoids 0 & log(0)

46  avi\_->adj\_ += adj\_ \* bd\_ \* val\_ / avi\_->val\_;

47  }

48  }

49  };

50

51  class pow\_dv\_vari : public op\_dv\_vari {

52  public:

53  pow\_dv\_vari(double a, vari\* bvi) :

54  op\_dv\_vari(std::pow(a,bvi->val\_),a,bvi) {

55  }

56  void chain() {

57  if (unlikely(boost::math::isnan(bvi\_->val\_)

58  || boost::math::isnan(ad\_)))

59  bvi\_->adj\_ = std::numeric\_limits<double>::quiet\_NaN();

60  else {

61  if (ad\_ == 0.0) return; // partials zero, avoids 0 & log(0)

62  bvi\_->adj\_ += adj\_ \* std::log(ad\_) \* val\_;

63  }

64  }

65  };

66  }

67

106  inline var pow(const var& base, const var& exponent) {

107  return var(new pow\_vv\_vari(base.vi\_,exponent.vi\_));

108  }

109

122  inline var pow(const var& base, const double exponent) {

123  if (exponent == 0.5)

124  return sqrt(base);

125  if (exponent == 1.0)

126  return base;

127  if (exponent == 2.0)

128  return base \* base; // FIXME: square() functionality from special\_functions

129  return var(new pow\_vd\_vari(base.vi\_,exponent));

130  }

131

144  inline var pow(const double base, const var& exponent) {

145  return var(new pow\_dv\_vari(base,exponent.vi\_));

146  }

147

148  }

149 }

150 #endif

boost::math::isnan

bool isnan(const stan::agrad::var &v)

Checks if the given number is NaN.

**Definition:** boost\_fpclassify.hpp:69

dv\_vari.hpp

stan::agrad::pow

fvar< T > pow(const fvar< T > &x1, const fvar< T > &x2)

**Definition:** pow.hpp:17

vd\_vari.hpp

stan::agrad::var::vi\_

vari \* vi\_

Pointer to the implementation of this variable.

**Definition:** var.hpp:40

unlikely

#define unlikely(x)

**Definition:** likely.hpp:9

stan::agrad::sqrt

fvar< T > sqrt(const fvar< T > &x)

**Definition:** sqrt.hpp:15

operator\_multiplication.hpp

var.hpp

sqrt.hpp

stan::agrad::var

Independent (input) and dependent (output) variables for gradients.

**Definition:** var.hpp:27

vv\_vari.hpp

stan::agrad::log

fvar< T > log(const fvar< T > &x)

**Definition:** log.hpp:15


---

[ Stan Home Page ]

*© 2011–2014,
Stan Development Team.*
